# Efficient visible luminescence of nanocrystalline silicon prepared from amorphous silicon films by thermal annealing and stain etching

**DOI:** 10.1186/1556-276X-6-349

**Published:** 2011-04-19

**Authors:** Victor Yur'evich Timoshenko, Kirill Alexandrovich Gonchar, Ivan Victorovich Mirgorodskiy, Natalia Evgen'evna Maslova, Valery Eduardovich Nikulin, Gaukhar Kalizhanovna Mussabek, Yerzhan Toktarovich Taurbaev, Eldos Abugalievich Svanbayev, Toktar Iskataevich Taurbaev

**Affiliations:** 1 Department of Physics, Lomonosov Moscow State University, Leninskie Gory 1, Moscow 119991, Russia; 2 al Farabi Kazakh National University, 96 Tole bi Str., Almaty 050000, Kazakhstan

## Abstract

Films of nanocrystalline silicon (nc-Si) were prepared from hydrogenated amorphous silicon (a-Si:H) by using rapid thermal annealing. The formed nc-Si films were subjected to stain etching in hydrofluoric acid solutions in order to passivate surfaces of nc-Si. The optical reflectance spectroscopy revealed the nc-Si formation as well as the high optical quality of the formed films. The Raman scattering spectroscopy was used to estimate the mean size and volume fraction of nc-Si in the annealed films, which were about 4 to 8 nm and 44 to 90%, respectively, depending on the annealing regime. In contrast to as-deposited a-Si:H films, the nc-Si films after stain etching exhibited efficient photoluminescence in the spectral range of 600 to 950 nm at room temperature. The photoluminescence intensity and lifetimes of the stain etched nc-Si films were similar to those for conventional porous Si formed by electrochemical etching. The obtained results indicate new possibilities to prepare luminescent thin films for Si-based optoelectronics.

## Introduction

Research interest in all silicon (Si)-based light-emitting device (LED) is stimulated by observation of efficient light emission in structures of nanocrystalline silicon (nc-Si) in SiO_2 _matrix (see for examples ref. [1]). Porous Si (por-Si) formed by electrochemical etching (anodization) of crystalline Si (c-Si) is a bright example of nc-Si-based material with rather high (up to 10%) quantum efficiency of the light emission under optical excitation or electrical current injection (see for example ref. [2]). Since por-Si based LEDs are characterized by low stability, the search of new materials, which are based on nc-Si and exhibit efficient and stable luminescence, is still belonging to actual tasks of modern technology and physics of low-dimensional structures.

Recently nc-Si films with efficient photoluminescence (PL) in the visible spectral range were formed by wet chemical etching, i.e. stain etching (SE), of microcrystalline Si deposited by plasma-enhanced chemical vapor deposition (PECVD) in silane highly diluted by hydrogen [3]. According to ref. [3], both as-deposited a-Si:H films and those after SE did not exhibit remarkable PL. This fact agrees with previously reported results [4]. While a-Si:H can be crystallized by using conventional furnace annealing (see for example ref. [4]) or rapid thermal annealing (RTA) [5], as-crystallized films are usually characterized by high density of non-radiative defects and do not demonstrate remarkable PL properties [5]. Furthermore, the furnace annealing (FA) of a-Si:H results in formation of polycrystalline Si films with rather large sizes of crystallites, which are typically far from the quantum confinement regime [4]. In the present article, we report the preparation of photoluminescent nc-Si films by using RTA or FA of a-Si:H films followed by SE treatments in HF-based solution.

## Experimental results and discussion

Films of a-Si:H with thickness of about 0.5 to 1 μm were deposited on quartz substrates by dc silane decomposition or magnetron sputtering (see for details ref. [6]). The deposited films were subjected to conventional RTA using irradiation by a tungsten halogen lamp with a single-pulse cycle to increase the temperature from room temperature to 900°C or to 950°C and back. The annealing was done in N_2 _flow. The total annealing time was varied from 10 to 50 s. Some parts of as-deposited a-Si:H films were thermally annealed in a furnace in vacuum conditions for 30 min at 950°C (see for details Table 1). The RTA and FA treated samples were then stain-etched by dipping them in the following mixtures: HF(40%):HNO_3 _= 100:1 for 3 to 5 s or in HF(40%):FeCl_3_:H_2_O = 1:2:2 for 30 min.

**Table 1:** Preparation parameters of the annealed samples and data on the mean size and volume fraction of nc-Si obtained from the Raman spectra analysis.

Sample	Annealing conditions			Mean size of nc-Si (nm)	Volume fraction of nc-Si to amorphous Si (%)
	Method	*T *(°C)	Duration		
#1	RTA	900	40 s	3.9	44
#2	RTA	950	50 s	5.0	88
#3	FA	950	30 min	8.0	90

The initial a-Si:H films and thermally annealed ones were investigated by means of the transmission electron microscopy (TEM), the reflection optical spectroscopy in the ultraviolet-visible-near-infrared (UV-VIS-NIR) region, Raman scattering and PL methods. The TEM analysis was done with a LEO 912 AB Omega electron microscope. A Perkin-Elmer Lambda 35 spectrophotometer was used for the reflectance measurements in the spectral range from 200 to 1100 nm. The Raman scattering was investigated by using a LabRAM HR 800 micro-Raman spectrometer with an Ar-laser at wavelength of 488 nm for the excitation. The PL was excited by a N_2_-laser (wavelength 337 nm, pulse duration 10 ns, repetition rate 100 Hz). The PL signal was dispersed by a 50-cm monochromator and was detected by a charge-coupled-device in the spectral range from 350 to 1100 nm. The PL transients were measured by a photomultiplier with a time resolution of about 100 ns. All optical studies were carried out at room temperature in air.

According to the TEM data (non shown), as-deposited a-Si:H films were amorphous and did not include voids or incorporations. The same films subjected to RTA or FA at temperatures about 900 to 950°C were found to consist of small (2 to 10 nm) nc-Si with volume fraction depended on the annealing time.

**Figure 1:**
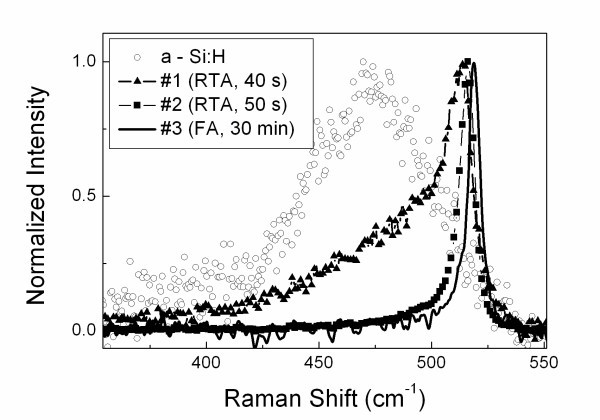
**Normalized Raman scattering spectra of a-Si:H film (circles) and samples #1 (triangles), #2 (squares), and #3 (solid line)**.

Figure 1 shows the Raman scattering spectra of as-deposited a-Si:H film and the annealed ones. The Raman spectrum of a-Si:H is represented by a broad line centered at 480 cm^-1^, which is typical for amorphous Si. The Raman spectra of the prepared nc-Si films exhibit a maximum at 513 to 519 cm^-1^, which is shifted to low frequency range in respect to the peak of c-Si substrate (520.5 cm^-1^). The shift is usually attributed to the phonon confinement in nc-Si [7]. The size of nc-Si and volume fraction to amorphous Si were estimated from the Raman spectra by using a method reported in ref. [8]. The results shown in Table 1 demonstrate that the RTA treatment with longer duration induced formation of nc-Si with larger mean size and volume fraction. Note, that the FA treatment for 30 min resulted in almost complete crystallization with the nc-Si size of about 8 nm.

**Figure 2:**
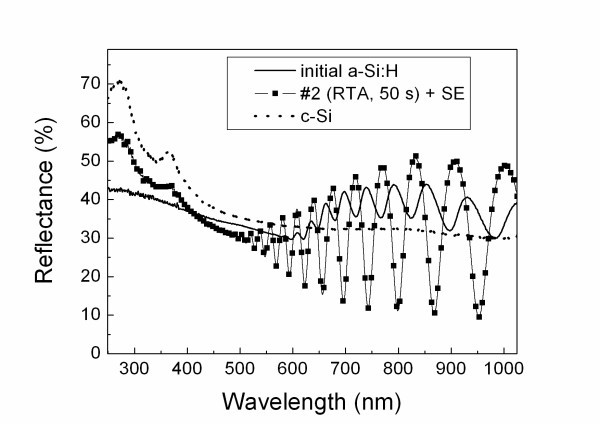
**Reflection spectra of a-Si:H film (solid line), sample #2 after SE (squares), and c-Si wafer (dotted line), for comparison**.

The reflection spectra of the prepared nc-Si films (see Figure 2) reveal two reflection bands at 274 and 368 nm, which are well known for c-Si and can be explained by the optical transitions near the direct band gap [2]. The interference patterns in the VIS-NIR ranges indicate the high optical quality of the films. The refractive indices of the films after RTA evaluated from the interference pattern can be explained by contributions of both amorphous and crystalline phases of Si. Note the quantitative analysis of the data is complicated because of unknown partial volume of pores formed in the films due to the annealing-induced hydrogen evolution. On the one hand, the SE procedure decreases the optical density of the RTA-treated films. This effect is evidently related to an increase of the film porosity because of the chemical dissolution of material. On the other hand, the refraction bands in the UV regions were always observed for the thermally annealed films independently of the duration and type of SE. This fact gives evidence of preferential dissolution of a-Si phase in the annealed samples.

**Figure 3:**
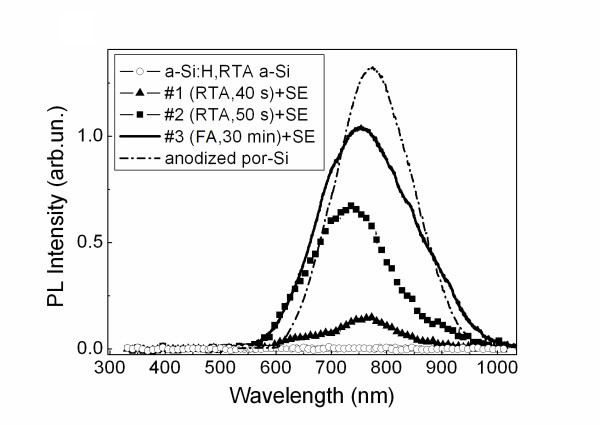
**Photoluminescence spectra of a-Si:H film and samples #1 (triangles), #2 (squares), and #3 (solid line) after SE**. The spectrum of porous Si prepared by electrochemical etching (anodization) is also shown for comparison.

The PL measurements did not reveal any remarkable light emission for as-prepared a-Si:H films as well as for the RTA-treated ones without the SE procedure. The efficient PL in the spectral range of 600 to 950 nm was observed for the thermally annealed films after the SE treatment (see Figure 3). The intensity, spectral position and lifetime of PL were similar to those measured for porous Si formed from c-Si by SE in the same solution or by standard anodization in HF-based electrolyte [2]. The observed PL of the nc-Si films after SE can be attributed to the radiative recombination of excitons confined in passivated nc-Si [9,10]. Since no PL was observed from the films of a-Si:H after SE the formation of nc-Si seems to be necessary for the efficient PL.

Figure 3 shows PL spectra of the annealed films after the SE treatment in HF:HNO_3 _solution. Similar spectra were obtained for another solution used for SE. The PL intensity increases for the films annealed for the longer time. The PL intensity of the annealed film after the SE treatment in HF:FeCl_3_:H_2_O solution was found to be two to three times higher than that for the sample treated in HF:HNO_3_. However the latter was characterized by better stability of the PL properties during illumination with excitation intensity above 100 W/cm^2 ^for several hours. Similarly to the PL of porous Si electrochemically grown in HF:HCl mixtures [11] this fact can be explained by better passivation of nc-Si surfaces because of the oxidizing effect of chlorine ions. The stronger PL intensity of the nc-Si films after SE in HF:FeCl_3_:H_2_O correlates with the Raman spectroscopy data, which indicate the presence of small nc-Si (see Figure 1 and Table 1). Both observations can be explained by slower rate of the chemical dissolution of nc-S/a-Si composite in HF:FeCl_3_-based solution in comparison with that for HF:HNO_3 _mixture. Also the stronger PL intensity of the samples treated in HF:FeCl_3_-based solution agrees with the results reported for microcrystalline Si films prepared by PECVD [3].

## Conclusion

The films of nc-Si with efficient PL were formed from a-Si:H by combining RTA and stain etching procedures and were investigated by means of the optical spectroscopy. An analysis of the Raman spectroscopy data showed that the mean size and volume fraction of nc-Si were depended on the preparation conditions. The obtained results demonstrate that the fast procedure of RTA followed by wet chemical etching can be used to obtain nc-Si-films with desired optical properties.

## Abbreviations

a-Si:H: hydrogenated amorphous silicon; FA: furnace annealing; LED: light-emitting device; nc-Si: nanocrystalline silicon; por-Si: porous Si; PL: photoluminescence; PECVD: plasma-enhanced chemical vapor deposition; RTA: rapid thermal annealing; SE: stain etching; TEM: transmission electron microscopy; UV-VIS-NIR: ultraviolet-visible-near-infrared.

## Competing interests

The authors declare that they have no competing interests.

## Authors' contributions

VYT carried out all the experiments and also initiated the first draft manuscript. KAG performed the photoluminescence measurements and assisted with the TEM data analysis. IVM provided the Raman spectra data from all samples. NEM participated in the Raman spectra analysis. VEN performed the optical reflection measurements. GKM contributed to the production of thin film. YTT performed the chemical treatment of the samples and provided the electrochemically prepared sample. EAS assisted in the thermal annealing of all samples and TIT contributed through interpretation of the experimental results and discussion of the manuscript. All authors read and approved the final manuscript.
